# Classifying perinatal mortality using verbal autopsy: is there a role for nonphysicians?

**DOI:** 10.1186/1478-7954-9-42

**Published:** 2011-08-05

**Authors:** Cyril Engmann, John Ditekemena, Imtiaz Jehan, Ana Garces, Mutinta Phiri, Vanessa Thorsten, Manolo Mazariegos, Elwyn Chomba, Omrana Pasha, Antoinette Tshefu, Elizabeth M McClure, Dennis Wallace, Robert L Goldenberg, Waldemar A Carlo, Linda L Wright, Carl Bose

**Affiliations:** 1 Departments of Pediatrics and Maternal Child Health, University of North Carolina at Chapel Hill, North Carolina, USA; 2 Kinshasa School of Public Health, Kinshasa, Democratic Republic of Congo; 3 Department of Community Health Sciences, The Aga Khan University, Karachi, Pakistan; 4 IMSALUD/San Carlos University, Guatemala City, Guatemala; 5 Department of Pediatrics and Child Health, University Teaching Hospital, Lusaka, Zambia; 6 Research Triangle Institute, Durham, North Carolina, USA; 7 Institute of Nutrition for Central America and Panama (INCAP), Guatemala City, Guatemala; 8 College of Medicine, Drexel University, Philadelphia, Pennsylvania, USA; 9 Department of Pediatrics, University of Alabama at Birmingham, Alabama, USA; 10 Eunice Kennedy Shriver National Institute of Child Health and Human Development, Bethesda, Maryland, USA

## Abstract

**Background:**

Because of a physician shortage in many low-income countries, the use of nonphysicians to classify perinatal mortality (stillbirth and early neonatal death) using verbal autopsy could be useful.

**Objective:**

To determine the extent to which underlying perinatal causes of deaths assigned by nonphysicians in Guatemala, Pakistan, Zambia, and the Democratic Republic of the Congo using a verbal autopsy method are concordant with underlying perinatal cause of death assigned by physician panels.

**Methods:**

Using a train-the-trainer model, 13 physicians and 40 nonphysicians were trained to determine cause of death using a standardized verbal autopsy training program. Subsequently, panels of two physicians and individual nonphysicians from this trained cohort independently reviewed verbal autopsy data from a sample of 118 early neonatal deaths and 134 stillbirths. With the cause of death assigned by the physician panel as the reference standard, sensitivity, specificity, positive and negative predictive values, and cause-specific mortality fractions were calculated to assess nonphysicians' coding responses. Robustness criteria to assess how well nonphysicians performed were used.

**Results:**

Causes of early neonatal death and stillbirth assigned by nonphysicians were concordant with physician-assigned causes 47% and 57% of the time, respectively. Tetanus filled robustness criteria for early neonatal death, and cord prolapse filled robustness criteria for stillbirth.

**Conclusions:**

There are significant differences in underlying cause of death as determined by physicians and nonphysicians even when they receive similar training in cause of death determination. Currently, it does not appear that nonphysicians can be used reliably to assign underlying cause of perinatal death using verbal autopsy.

## Background

Understanding population-based causes of perinatal death (stillbirth [SB] and early neonatal deaths [END], i.e., newborn deaths in the first seven days of life) is essential when developing an effective perinatal health policy [1]. Because there will always be competing demands for health care resources, a robust system constructed to identify and assign a medically-determined cause of death (COD) for each perinatal death is highly desirable [2]. In many high-income countries, there is a complete record of each death, and 90% of these have medical certification of COD [3]. By contrast, many low- and middle- income countries, which have the highest burden of poverty and disease, continue to lack routine, representative, and high-quality information on COD and population-based cause-specific mortality fractions (CSMF) [4]. Fewer than 3% of all perinatal deaths in low- and middle-income countries have medical certification of COD [5]. In part, this may be because more than half of all births and perinatal deaths occur in the home and are frequently unrecorded in vital registration or health systems [6].

Increasing numbers of low- and middle- income countries are using verbal autopsy (VA) methods as an epidemiologic tool to inform mortality surveillance systems [7]. To determine perinatal mortality, the VA method relies on information obtained from an interview with the primary caregiver (usually the mother) of the deceased. During this process, the symptoms, signs, and behaviors during the illness of the deceased, or of the mother in the case of fetal death, are recorded [8]. This information is summarized and reviewed and the most probable COD assigned. VA is proving to be a cost-effective, practical, and sustainable alternative to a thorough medical diagnostic evaluation where vital registration systems are weak [9].

A variety of methods exist for interpreting VA interviews to arrive at a COD. The most commonly used method has two or three trained physician coders review the data and independently assign a COD [10]. Any discrepancies between the COD assigned by each physician member of the panel are resolved by discussion and review of the VA data, and a final consensus COD is agreed upon by the physician panel. Alternatively, COD can be assigned by the use of predetermined criteria/algorithms, computer simulations, or probabilistic approaches, all of which do not require the presence of a physician [11-15].

There is a widespread physician shortage in many low-income countries and significant costs incurred in recruiting, training, and utilizing physicians. Reports suggest that nonphysician providers can conduct specified clinical tasks with adequate training [16-18]. We previously reported that when taught a standardized VA package in a classroom setting, nurses and midwives achieve a level of cognitive and applied knowledge comparable to physicians in determining perinatal COD [19]. Thus, we sought to investigate whether, following this training, nonphysicians can determine causes of SB and END in rural communities as reliably as physicians.

## Methods

### Setting, subjects, and study design

This prospective observational study was nested within the FIRST BREATH Trial conducted by the Eunice Kennedy Shriver National Institute of Child Health and Human Development Global Network for Women's and Children's Health Research [20]. The FIRST BREATH Trial was a cluster randomized, controlled trial that investigated the effects of implementing a package of newborn care practices and newborn resuscitation in community settings.

This VA study included 38 communities from Guatemala (Chimaltenango province), the Democratic Republic of the Congo (DRC) (Equateur province), Zambia (Kafue district), and Pakistan (Thatta district). Each community comprised a cluster of villages with approximately 300 deliveries per year. Data describing births were collected by birth attendants and reviewed by trained nurse-midwives (with three to four years of health training) or community health workers (high school graduates with 18 months of health training) designated as community coordinators. Within one week of an END or SB, birth attendants notified community coordinators who then visited the family, determined eligibility for the study, and requested consent from eligible mothers. Perinatal deaths were excluded if they occurred in a hospital, if a birth attendant was not present at delivery, if the mother was unavailable for any reason (including peripartum death), or if the mother could not be enrolled within seven days of death. A seven-day enrollment window was chosen to reduce the variability in the quality of reporting introduced by recall bias [21-23]. Because the conventional perinatal VA respondents are mothers, we included only those subjects whose mothers were available for interview. Informed consent was obtained from mothers in a private and confidential setting. The consent form was read to all mothers who then provided their signatures or, if they were illiterate, thumbprints.

### Training and VA methodology

Neither community coordinators nor physicians had prior experience before the study with the use of VA to determine COD. All community coordinators and physicians participating in this study received standardized training in VA methods over three days, via a train-the-trainer method [19]. Community coordinators were trained to interview mothers using the VA questionnaire. To assign underlying COD, both community coordinators and physicians were trained in the classification, rules, and guidelines of the 10th revision of the International Classification of Diseases (ICD-10). Underlying COD was defined as the single most important disease or condition that initiated the train of morbid events leading directly to fetal or neonatal death.

Uniform data describing the circumstances surrounding a perinatal death were collected from each mother using a standardized VA questionnaire developed specifically for this study from a validated VA tool [24]. The questionnaire was administered by the community coordinators who then sent these data separately to two local physicians. Additionally, the community coordinators and physicians were provided with demographic and other descriptive data collected as part of the FIRST BREATH Trial. Each community coordinator and physician independently first determined whether the death occurred prior to birth and was therefore classified as a SB, or after a live birth and classified as an END. Then they assigned one underlying COD. After the COD was assigned and entered independently, any discrepancy in assignment of COD between physicians was discussed and consensus underlying COD was assigned. The underlying COD assigned by the community coordinator was then compared to the consensus underlying COD assigned by the physician panel.

### Data collection and analysis

Data were entered and transmitted electronically to the data coordinating center (Research Triangle Institute, Research Triangle Park, NC, USA) where data edits, including inter- and intraform consistency checks, were performed. The study was reviewed and approved by the institutional ethics review committees of the Research Triangle Institute, the University of North Carolina at Chapel Hill, and in-country Institutional Review Boards.

Data were analyzed using SAS (SAS/STAT^® ^Software version 9.2). Physician perinatal COD responses were viewed as the reference standard for calculations of sensitivity, specificity, positive and negative predictive values, and CSMF, which were calculated using conventional two-by-two table analysis. The Delta method was used to calculate confidence intervals for the CSMFs  [25]. We defined the CSMF as the number of perinatal deaths (END or SB) due to a specific cause divided by the total number of deaths.

Before the start of the study, our a priori hypothesis was that the COD assigned by community coordinators would be concordant with the COD assigned by the physician panel in greater than 70% of perinatal deaths, and we powered our study accordingly. We also assessed the degree of robustness of community coordinator responses. We defined robustness using criteria previously described, utilized, and published by Setel et al [26]. To be considered "robust" a condition must meet the following criteria: 1) sensitivity > 50%, 2) specificity > (1-CSMF of the physician consensus), and 3) relative difference between the CSMF for the community coordinator and the CSMF for the physician consensus within 20%. The relative difference was calculated as follows: absolute value ((CSMF of the physician consensus - CSMF of the community coordinator)/CSMF of the physician consensus × 100%). Additionally, we calculated the level of agreement between the physician consensus and community coordinators, using Cohen's kappa statistic. Levels of agreement based on ranges of kappa values were defined as follows: 0.81-0.99, almost perfect agreement; 0.61-0.80, substantial agreement; 0.41-0.60 moderate agreement; and less than 0.4, slight to fair agreement [24].

## Results

**Figure 1:**
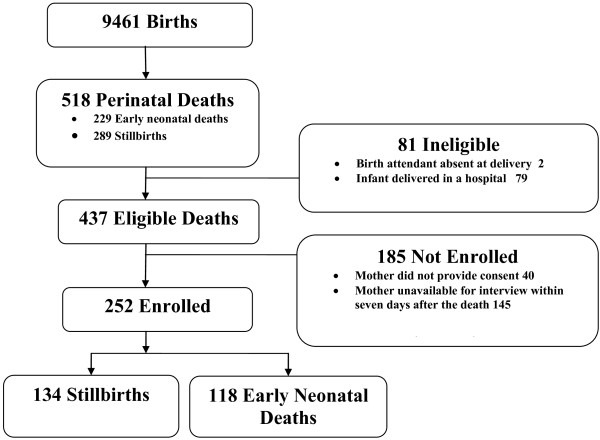
**The verbal autopsy study population**.

The study period was from May 2007 to June 2008, during which 9,461 infants were born in the designated communities. Among these, birth attendants identified 518 SB and END (Figure 1). The SB, END, and perinatal mortality rates were 30/1000 births, 25/1000 live births, and 55/1000 births, respectively. Of the 518 deaths, 81 were ineligible for the study because the delivery occurred in a hospital (79) or the birth attendant was absent at the time of delivery (2). Among eligible deaths, 185 were not enrolled because the mother was not available for interview within seven days after the death (145) or did not provide consent (40). This study reports on 252 perinatal deaths (134 SBs and 118 ENDs), based on determinations by the physicians regarding the timing of perinatal deaths.

### Concordance of stillbirth and early neonatal death between physicians and nonphysicians

Ninety-three percent of perinatal deaths determined by physicians to be SBs were classified as SBs by community coordinators; the remainder were classified as ENDs. Ninety-five percent of perinatal deaths determined by physicians to be ENDs were classified by community coordinators as ENDs; the remainder were classified as SBs. Concordance between physicians and community coordinators in the determination of timing of perinatal deaths did not vary between the two classes of community coordinators (nurse-midwives and community health workers).

### Early neonatal death

Table 1 compares underlying causes of END assigned by physician panels and community coordinators. Overall, causes of END assigned by community coordinators were concordant with causes of END assigned by physician panels 47% of the time. Table 2 describes the sensitivity, specificity, positive and negative predictive values, and CSMF of specific underlying causes of END assigned by community coordinators. Kappa values are additionally included. Sensitivity and specificity were high for preterm/low birth weight and tetanus. By contrast, sensitivity was low for infections and asphyxia, although specificity for both of these was 0.90 or above. The positive predictive values for infections and tetanus were 0.83 and 0.67, respectively, and the negative predictive values for preterm/low birth weight, trauma, and tetanus were 0.93 or above. The relative difference between CSMF assigned by physician panels and community coordinators was 20% for tetanus and > 20% for all other diagnostic categories. Only the diagnosis of tetanus fulfilled criteria for robustness. When the level of agreement among the different diagnostic categories was considered using Cohen's kappa statistic, there was substantial agreement for the diagnosis of tetanus (0.71); all other categories showed slight or only moderate agreement.

**Table 1:** Comparison of physician consensus (PC) and community coordinator (CC) for underlying neonatal cause of death (COD)

CC for underlying cause of death	PC for underlying cause of death							CC total
		
	Preterm/ Low birth weight		Asphyxia	Fetal Trauma	Tetanus	Unknown/ no cause	**Other**^**1**^	
Preterm/low birth weight	**14**	11	3	0	1	0	2	31

Infection	1	**19**	3	0	0	0	0	23

Asphyxia	4	5	**13**	0	0	0	0	22

Fetal trauma	0	4	3	**0**	0	0	0	7

Tetanus	0	2	0	0	**4**	0	0	6

Unknown/no cause	0	6	3	0	0	**2**	1	12

Other^1^	1	5	6	0	0	2	**3**	17

**PC total**	20	52	31	0	5	4	6	118

^1^Other includes congenital malformation, birth trauma, and neonatal accident,

Percent concordant = (55/118) × 100 = 46.6%. (This includes COD coded as Other.)

**Table 2:** Comparison of neonatal underlying cause reported by physician consensus (PC) to underlying cause of death reported by community coordinator (CC) for select causes of early neonatal death (n = 118)

Underlying COD, as determined by CC	Underlying COD, as determined by PC (reference standard)		**Measures**^**2**^									
	
	Identified as COD	Not identified as COD	SE	SP	PPV	NPV	CSMF_PC_ (95% CI)	1-CSMF_PC_	CSMF_CC_ (95% CI)	1-CSMF_CC_	RD (95% CI)	Kappa (95 CI)
Preterm/LBW	20	98	0.70	0.83	0.45	0.93	0.17 (0.09, 0.25)	0.83	0.26 (0.16, 0.37)	0.74	0.55 (0.08,1.04)	0.43 (0.24, 0.62)
COD	14 (70.0)	17 (17.3)										
Not COD	6 (30.0)	81 (82.7)										

Infection	52	66	0.37	0.94	0.83	0.65	0.44 (0.29, 0.59)	0.56	0.19 (0.11, 0.28)	0.81	0.56 (0.40, 0.70)	0.32 (0.17, 0.48)
COD	19 (36.5)	4 (6.1)										
Not COD	33 (63.5)	62 (93.9)										

Asphyxia	31	87	0.42	0.90	0.59	0.81	0.26 (0.16, 0.37)	0.74	0.19 (0.10, 0.27)	0.81	0.29 (0.03, 0.54)	0.35 (0.15, 0.54)
COD	13 (41.9)	9 (10.3)										
Not COD	18 (58.1)	78 (89.7)										

Fetal Trauma	0	118	--	0.94	0.00	1.00	--	--	0.06 (0.01, 0.11)	0.94	--	
COD	0 (0.0)	7 (5.9)										
Not COD	0 (0.0)	111 (94.1)										

Tetanus	5	113	0.80	0.98	0.67	0.99	0.04 (0.00, 0.08)	0.96	0.05 (0.01, 0.09)	0.95	0.20 (0.00,2.00)	0.71 0.41, 1.00)
COD	4 (80.0)	2 (1.8)										
Not COD	1 (20.0)	111 (98.2)										

Unknown/ no cause	4	114	0.50	0.91	0.17	0.98	0.03 (0.00, 0.07)	0.97	0.10 (0.04, 0.16)	0.90	2.00 (0.27, 11.0)	0.21 (-0.07, 0.49)
COD	2 (50.0)	10 (8.8)										
Not COD	2 (50.0)	104 (91.2)										

Other^1^	6	112	0.50	0.88	0.18	0.97	0.05 (0.01, 0.09)	0.95	0.14 (0.07, 0.22)	0.86	1.83 (0.38,7.33)	0.20 (-0.04, 0.44)
COD	3 (50.0)	14 (12.5)										
Not COD	3 (50.0)	98 (87.5)										

^1 ^Other includes congenital malformation, birth trauma, and neonatal accident.

^2 ^Definitions of measures are provided in the methods section. Measures are as follows: sensitivity (SE), specificity (SP), positive predictive value (PPV), negative predictive value (NPV), cause-specific mortality fraction (CSMF) of physician consensus (PC) and community coordinator (CC), and absolute relative difference of the CSMF (RD). RD was calculated using the actual numeric values rather than the rounded CSMFs reported in the table.

### Stillbirth

Table 3 summarizes the comparison of community coordinator and physician underlying COD for SB. Overall, causes of SB assigned by community coordinators were concordant with causes of SB assigned by physician panels 57% of the time. Table 4 presents the sensitivity, specificity, positive and negative predictive values, and CSMF of underlying SB COD assigned by community coordinators. Kappa values are additionally included. Sensitivity for antepartum hemorrhage and maternal infection was 0.62 and 0.64, respectively, and for all other known COD categories was 0.57 or less. Specificity was generally high (0.94 or higher) except for prematurity, for which the specificity was 0.86. The positive predictive values of antepartum hemorrhage, maternal infection, maternal accident, and prolonged labor were 0.80 or higher. Maternal infections had a negative predictive value of 0.81; all the other SB COD categories had values of 0.94 or above. The relative difference between CSMF assigned by physician panels and community coordinators was 13% for cord prolapse; all other SB diagnostic categories were greater than 20%. Cord prolapse fulfilled the robustness criteria. It is worth pointing out that where relative difference values are small, it may be because there are very small CSMFs. When the level of agreement among the different diagnostic categories was considered using Cohen's kappa statistic, no category demonstrated almost perfect agreement.

**Table 3:** Comparison of physician consensus (PC) and community coordinator (CC) for underlying maternal cause of stillbirth

CC for underlying maternal cause of death	PC underlying maternal cause of death									CC total
		
	Antepartum hemorrhage		Prematurity	Accident	Prolonged labor	Cord prolapse/complication	Malpre-sentation	Unknown/no cause	**Other**^**1**^	
Antepartum hemorrhage	**8**	1	0	0	1	0	0	0	0	10

Maternal infection/sepsis	1	**32**	0	0	0	1	0	2	1	37

Prematurity	3	9	**4**	1	0	0	0	2	2	21

Maternal accident	0	1	0	**4**	0	0	0	0	0	5

Prolonged labor	0	0	0	0	**8**	0	2	0	0	10

Cord prolapse/complication	1	2	0	0	2	**4**	0	0	0	9

Malpresentation	0	1	0	0	0	0	**2**	0	2	5

Unknown/no cause	0	3	5	2	1	1	0	**11**	4	27

Other^1^	0	1	0	0	3	2	0	1	**3**	10

**PC total**	13	50	9	7	15	8	4	16	12	134

^1 ^Other includes multiple delivery, hypertension/eclampsia, and post-term delivery

Percent concordant = (76/134) × 100 = 56.7%. (This includes COD coded as Other.)

**Table 4:** Comparison of maternal underlying cause reported by physician consensus (PC) to underlying cause reported by community coordinator (CC) for select causes of stillbirth (n = 134)

Underlying COD, as determined by CC	Underlying COD, as determined by PC (reference standard)		**Measures**^**2**^									
	
	Identified as COD	Not identified as COD	SE	SP	PPV	NPV	CSMF_PC_ (95% CI)	1-CSMF_PC_	CSMF_CC_ (95% CI)	1-CSMF_CC_	RD (95% CI)	Kappa (95% CI)
Antepartum hemorrhage	13	121	0.62	0.98	0.80	0.96	0.10 (0.04, 0.15)	0.90	0.07 (0.03, 0.12)	0.93	0.23 (0.00,0.57)	0.67 (0.44,0.9)
COD	8 (61.5)	2 (1.7)										
Not COD	5 (38.5)	119 (98.3)										

Maternal infection	50	84	0.64	0.94	0.86	0.81	0.37 (0.25, 0.50)	0.63	0.28 (0.17, 0.38)	0.72	0.26 (0.87,0.42)	0.62 (0.47,0.75)
COD	32 (64.0)	5 (6.0)										
Not COD	18 (36.0)	79 (94.0)										

Prematurity	9	125	0.44	0.86	0.19	0.96	0.07 (0.02, 0.11)	0.93	0.16 (0.08, 0.23)	0.84	1.33 (0.24,4.40)	0.19 (-0.02,0.4)
COD	4 (44.4)	17 (13.6)										
Not COD	5 (55.6)	108 (86.4)										

Maternal accident	7	127	0.57	0.99	0.80	0.98	0.05 (0.01, 0.09)	0.95	0.04 (0.00, 0.07)	0.96	0.29 (0.00,0.80)	0.65 (0.33,0.97)
COD	4 (57.1)	1 (0.8)										
Not COD	3 (42.9)	126 (99.2)										

Prolonged labor	15	119	0.53	0.98	0.80	0.94	0.11 (0.05, 0.17)	0.89	0.07 (0.03, 0.12)	0.93	0.33 (0.00,0.64)	0.6 (0.37,0.84)
COD	8 (53.3)	2 (1.7)										
Not COD	7 (46.7)	117 (98.3)										

Cord prolapse/complication	8	126	0.50	0.96	0.44	0.97	0.06 (0.02, 0.10)	0.94	0.07 (0.02, 0.11)	0.93	0.13 (0.00,1.67)	0.43 (0.13,0.74)
COD	4 (50.0)	5 (4.0)										
Not COD	4 (50.0)	121 (96.0)										

Malpresentation	4	130	0.50	0.98	0.40	0.98	0.03 (0.00, 0.06)	0.97	0.04 (0.00, 0.07)	0.96	0.25 (0.00,3.00)	0.42 (0.01,0.84)
COD	2 (50.0)	3 (2.3)										
Not COD	2 (50.0)	127 (97.7)										

Unknown/no cause	16	118	0.69	0.86	0.41	0.95	0.12 (0.06, 0.18)	0.88	0.20 (0.12, 0.29)	0.80	0.69 (0.12,1.83)	0.43 (0.23,0.62)
COD	11 (68.8)	16 (13.6)										
Not COD	5 (31.3)	102 (86.4)										

Other^1^	12	122	0.25	0.94	0.30	0.93	0.09 (0.04, 0.14)	0.91	0.07 (0.03, 0.12)	0.93	0.17 (0.00,0.82)	0.21 0.05,0.47)
COD	3 (25.0)	7 (5.7)										
Not COD	9 (75.0)	115 (94.3)										

^1 ^Other includes multiple delivery, hypertension/eclampsia, post-term delivery, and other causes.

^2 ^Definitions of measures are provided in the methods section. Measures are as follows: sensitivity (SE), specificity (SP), positive predictive value (PPV), negative predictive value (NPV), cause-specific mortality fraction (CSMF) of physician consensus (PC) and community coordinator (CC), and absolute relative difference of CSMF (RD). RD was calculated using the actual numeric values rather than the rounded CSMFs reported in the table.

## Discussion

There are three main findings from this study. The first is that given identical data from the VA questionnaires, community coordinators and physicians draw the same conclusions about the timing of perinatal death (SB and END) 95% of the time. Second, causes of SB and END assigned by community coordinators were concordant with causes of SB and END assigned by physician panels 57% and 47% of the time, respectively. Third, only one cause of END assigned by community coordinators (tetanus) met robustness criteria. Similarly, when robustness criteria were applied to SB diagnostic categories to assess the performance of community coordinators, only cord prolapse met criteria.

Task-shifting of physician-domain responsibilities is an increasingly important concept that is gaining support in the literature [16,17]. Numerous authors have assessed the utilization and impact of nonphysician providers after being taught a structured curriculum [18,19]. These authors report that nonphysicians, specifically nurse-midwives, can perform comparably to physicians when taught a structured teaching program with adequate supervision. To our knowledge, only one study has compared nonphysicians to physicians in determining perinatal COD in the field using verbal autopsy methods, and none has compared nonphysicians from a variety of countries with a range of backgrounds such as nurse-midwives in Zambia and DRC and community health workers in Pakistan and Guatemala [27]. Our group previously examined how well community coordinators and physicians performed when taught a structured VA program in a classroom setting [19]. In both cognitive and applied knowledge, community coordinators' pretest results were lower than physicians; however, these results improved significantly post-test, with nurse-midwives showing comparable results to physicians. In light of these data we undertook the present study. Our study showed that despite the ability to improve cognitive and applied knowledge in the classroom setting, this knowledge did not result in nonphysicians reaching similar conclusions about COD in actual practice.

Chowdhury et al. reported on the use of medical assistants (with three years of institutional training) in a single site in Matlab, Bangladesh, to interpret neonatal VA data and assign COD [27]. When specific diagnostic categories assigned by medical assistants were compared to physician panels, birth asphyxia showed good reliability with kappa values of 0.77, while prematurity, respiratory distress syndrome, pneumonia, and sepsis/meningitis showed moderate agreement, with kappa values between 0.51 and 0.59. The authors concluded that medical assistants are generally knowledgeable about the disease profile of a geographic area, can generally use their clinical judgment and knowledge to determine COD for all ICD-10 classes of neonatal death, and may be considered an alternative for determining neonatal COD in rural areas where physicians are scarce. A number of reasons may explain the differences observed between our study and those of Chowdhury et al. First, in our study causes of both SB and END were assigned, in contrast to neonatal outcomes only from Matlab. Also, community coordinators had different educational backgrounds. Nurses and midwives had three to four years post-high school health training, whereas community health workers had only 18 months post-high school health training, in contrast to medical assistants from Matlab who had three years training. The community coordinators in our study had no a priori experience with VA and assigning COD, unlike the Matlab cohort, and our study was a multisite study compared to the single site of Matlab, where the medical assistants over the years have been closely involved in verbal autopsy work in the demographic surveillance program.

The concept of underlying COD, the single most important disease or condition that initiated the chain of events leading directly to fetal or neonatal death, is complex. It requires a deep appreciation of pathophysiology and, especially in the case of perinatal death, consideration of both the mother and the fetus. To effectively utilize this concept, the coder has to "construct a story" of what happened. The key initiating factor, without which the death most likely might not have occurred, is the cornerstone of the "story." It is possible that community coordinator responses might reflect other categories of COD, such as the final and contributing COD as described in the ICD-10. Further research is needed to determine whether concordance of community coordinator-assigned COD when utilizing multiple or other categories of COD might yield higher concordance with physician panels.

Comparing only underlying COD may be a potential limitation in this study. It appears increasingly evident that individual perinatal deaths in low-income countries may have several causes. Thus, forcing assignment of a perinatal death into a single underlying cause, as required for ICD-10, may be less useful than previously appreciated [14,28]. For example, a combination of prematurity, birth asphyxia, and infection may coexist in an END, and it may be more useful from a public health policy perspective to consider all causes of death collectively. Additionally, some authors use final COD instead of underlying COD assignments [27].

Although the use of nonphysician coders to assign COD may not be a suitable alternative to the use of physician coders, other alternatives may have a role. A number of computer simulation techniques have been developed that address multiple COD and CSMF. Byass et al. have described a Bayesian approach called InterVA that simultaneously adjusts the probability of a finite list of causes according to affirmative answers to specific symptoms [29]. This approach calculates the likelihood of each COD and displays as many as three of the most probable COD, along with their associated likelihoods [11]. More recently, King and Lu developed an alternative probabilistic method which directly estimates CSMF without individual COD attribution [1]. Data on symptoms reported by caregivers along with COD are collected, and the COD distribution is estimated in the population in which only symptom data are available. Each of these methods has its advantages and drawbacks in terms of cost effectiveness, complexity, repeatability, and validity. For example, the King-Lu method depends on the availability of high-quality, facility-based, or valid mortality data, which are lacking in most settings where VA is needed.

A major strength of this study is the use of a standardized VA training program for both nonphysicians and physicians. There are some limitations to this study. We did not use the harmonized VA questionnaire developed and published by WHO, since this was published after the start of the study. However, we believe that the results of our study would have been similar had we used this tool because the tools are broadly similar. Other limitations of this study include the lack of available medical diagnostic aids (laboratory, radiologic, or microbiologic studies) and lack of a postmortem examination for validating the underlying COD assigned by physicians. Although the COD determined by the physician panel is often the traditional reference standard in VA methodology, physician panels have their limitations: their assignments of COD may contain systematic biases, they may not readily code diseases unexpected in certain demographic groups, they tend to focus on the presence rather than the absence of symptoms, and they show a preference for highly specific diagnosis [12,30,31]. It is conceivable that direct interactions between the community coordinator and the respondents (mothers and birth attendants) may have provided the community coordinators with more information from the respondents and the environment about the circumstances, signs, and symptoms of the deceased before death than was recorded on the standardized VA questionnaire.

Verbal autopsy has been used in a variety of ways, including: to determine priority diseases and programmatic intervention; to conduct rapid assessments in emergency/disaster situations; as sample registration of vital events; and perhaps most importantly, to describe population-level CSMF. If Millennium Development Goals related to pregnancy outcomes are to be achieved, it is imperative to understand more about the perinatal CODs which contribute disproportionately to under-5 mortality in low-income countries. The shortage of human and material resources makes routine perinatal autopsies for deaths that occur in a community setting in low-income countries unlikely. The relatively limited number of symptoms and signs exhibited by the fetus and neonate compared to adults and the strength of studies conducted make perinatal verbal autopsy an attractive medium-term alternative.

## Conclusions

The most common method of assigning COD is the use of physician panels, which are costly and utilize scarce physician availability. In this study, the COD assigned by nonphysicians agreed with the COD determined by physicians about 50% of the time, and only tetanus and cord prolapse met robustness criteria. Although it may be too early to recommend against using nonphysicians to determine perinatal COD, based on our data, we recommend that further research be performed before nonphysicians are asked to determine perinatal COD in any settings in low-income countries.

## Competing interests

The authors declare that they have no competing interests.

## Authors' contributions

CE, VT, EM, DW, CB, WC, RG and LL had significant intellectual input in the conception and design of the study, data acquisition and analyses, draft writing, and final approval of the study. AG, IJ, JD, MP, MM, EC, OP and AT had significant input in the design of this study, data acquisition and analyses, draft writing, and final approval of the study. All authors read and approved the final manuscript.

## Funding

Funding was provided by grants from the National Institutes of Child Health and Human Development (U01 HD 40636) and the Bill & Melinda Gates Foundation.
